# The severity of distal sensory polyneuropathy increasing with HIV/AIDS stage

**DOI:** 10.11604/pamj.2024.48.51.33972

**Published:** 2024-06-07

**Authors:** Andi Weri Sompa, Yudy Goysal, Muhammad Akbar, Andi Dian Diarfah

**Affiliations:** 1 Neurology Department, Faculty of Medicine and Health Sciences, Universitas Muhammadiyah Makassar, Makassar, Indonesia,; 2 Neurology Department, Faculty of Medicine, Hasanuddin University, Makassar, Indonesia,; 3 Faculty of Medical and Health Sciences, UIN Alauddin, Makassar, Indonesia

**Keywords:** Distal sensory polyneuropathy (DSP), DSP severity, HIV/AIDS, neurological problem

## Abstract

Distal sensory polyneuropathy (DSP) is the most common neurological problem in HIV/AIDS Patients. It represents a complex symptom that occurs because of peripheral nerve damage related to advanced HIV disease and in association with the use of antiretroviral therapy. DSP is a frequent symptom in which the specific pathophysiology is not well understood. Recently, mitochondrial toxicity and antiretroviral toxic neuropathies have been more identified as a possible etiology of DSP. This study’s objective was to determine factors associated with DSP severity in HIV/AIDS patients. This cross-sectional study was followed by 50 HIV/AIDS outpatients at some hospitals in Makassar, Indonesia who met the inclusion criteria. DSP is diagnosed using non-invasive screening tools subjective peripheral neuropathy screen (SPNS) which can determine the severity of DSP in advance. Some factors were analyzed by using Pearson’s chi-square test and Spearman’s correlation test. Forty-three participants (86%) had diagnosed DSP which is mostly moderate in severity (48%). Statistical analysis showed significant correlation between HIV/AIDS Stage and DSP severity (p=0.032) meanwhile CD4 count, antiretroviral, body mass index (BMI), and hemoglobin level have no significant correlation to DSP severity. In conclusion, HIV/AIDS stage and DSP severity correlate where the later the stage the more severe DSP.

## To the editors of the Pan African Medical Journal

HIV infection is often accompanied by neurological disorders in the advanced phase [1]. With HIV cases increasing, it is increasingly clear that peripheral nerve disorders are increasing. Currently, HIV together with diabetes and leprosy are the three main causes of neuropathy worldwide. Among the various manifestations of peripheral nerve disorders in people with HIV, distal sensory polyneuropathy (DSP) is the highest manifestation of incidence rate and is the main cause of morbidity in HIV and AIDS patients [2]. This study's objective was to determine factors associated with DSP severity in HIV/AIDS patients. This cross-sectional study was followed by 50 HIV/AIDS outpatients at some hospitals in Makassar, Indonesia who met the inclusion criteria. The participants were all HIV outpatients taken on a non-probability sampling basis with consecutive methods that met the inclusion criteria. By subjective peripheral neuropathy screen (SPNS), subjects were determined whether DSP is experienced as well as the degree of its severity.

DSP is the most common neurological complication in HIV/AIDS infections [2,3]. The incidence of DSP in this study reached 86% (43 out of 50 sufferers) with various degrees, where the most experienced were moderate degree (48%), severe DSP experienced by 10 people (20%) and mild DSP experienced by 9 participants (18%). This figure (86%) is quite high, probably because most of the study subjects are patients who have experienced symptomatic neuropathy while in previous studies more are outpatients who are still in asymptomatic condition, here the pathology process runs slowly, and cell damage occurs subclinically (silent). This study also involves all stages whether using ARV or not. It has been known that DSP can occur at any stage of HIV/AIDS and 38% to 90% of HIV-DSP patients reported having neuropathic pain [2].

In this study, we analyze some factors associated with DSP and the severity of DSP. After statistical analysis, a positive correlation was shown between HIV stage and the severity of DSP (p= 0.032) (Table 1). This means that the later the HIV stage, the more severe of DSP. It was implied that the stage of infection has a strong relationship to sensory neuropathy in HIV. In Kietrys study [4], more DSP manifestation were symptomatic and affecting quality of live prominently because of neuropathic pain. In study by Robinson-Papp *et al*. [5] reported symptoms consistent with HIV neuropathy like tingling sensation, burning and numbness at lower extremities.

**Table 1:** distal sensory polyneuropathy by risk factors

Risk Factors	Sensory Disturbance		R	P
	DSP	Normal		
**Stadium HIV**				
1^st^ Stadium	5	3	0.264	0.032
2^nd^ Stadium	18	3		
3^rd^ Stadium	17	0		
4^th^ Stadium	3	1		
**CD4 level**				
< 200	31	4		
200-500	8	3	-0.115	0.212
> 500	4	0		
**ARV**				
Using ARV	18	3	-0.024	0.434
No ARV	25	4		
**Body Mass Index**				
Underweight	16	3	0.015	0.459
Normal	21	4		
Overweight	6	0		
**Hemoglobin**				
Normal	28	4	-0.005	0.487
Abnormal	15	3		

Meanwhile, this study showed that CD4 count, antiretroviral, body mass index (BMI), and hemoglobin level have no significant correlation to DSP severity (Table 1). In contrast to Imran *et al*. [6] who stated a meaningful relationship between CD4 and DSP levels, in this study CD4 levels were negatively correlated but meaningless to the degree of DSP, but the relationship between CD4 levels and HIV stage can indicate the influence of CD4 levels on the incidence and severity of DSP degrees because low CD4 levels indicate advanced infections. This was also reinforced by an analysis of the correlation between CD4 and HIV infection stages that showed a correlation (p < 0.01) (Table 1).

The use of ARV in this study had a negative correlation with the incidence and severity of DSP means that ARV users had a higher degree of DSP than DSP in HIV patients who did not use ARV (Table 1). It has been known that the use of ARV in the mid-1990s has successfully decreased the morbidity and mortality of people with HIV/AIDS. Life expectancy is also getting better. However, this is not the case with neurological complications that increase due to the neurotoxic effects of NRTI-group ARV such as stavudine, didanosin, and zalcitabin. Currently estimated DSP due to ARV occurs in 20% to more than 50% of cases [7,8].

BMI factors in this study were positively correlated but meaningless to the severity of DSP degrees (Table 1). BMI was associated with a person's nutritional status, and it was considered that poor nutritional status was characterized by less than normal BMI; resulting in various metabolic complications including deficiency of various nutrients especially vitamin B12. This vitamin known as neurotropic agent when in low levels causes demyelinating and myelin loss in the peripheral nerves [9,10]. Hemoglobin factor has also been widely researched. In this study, we found negative correlation (p > 0.05) (Table 1). According to Ances *et al*. [8] low hemoglobin levels are associated with advanced stages so can indirectly be related to the incidence of DSP and the severity of DSP degrees. A study by Imran *et al*. [6] also found a relationship to reflex disorders.

The incidence of DSP is 86% based on SPNS in this study. Some factors have been analyzed and only HIV /AIDS stage show positive correlation to the severity of DSP, in contrary with CD4 levels, ARV, BMI and hemoglobin levels.
